# Parkinsonian signs and cognitive trajectories in older adults: a population-based longitudinal study

**DOI:** 10.1093/ageing/afag160

**Published:** 2026-06-08

**Authors:** Charlotte Algotsson, Marieclaire Overton, Rani Basna, SöLve ElmstÅHl, Arkadiusz Siennicki-Lantz

**Affiliations:** Division of Geriatric Medicine, Department of Clinical Sciences in Malmö, Lund University, Malmö, Skåne County, Sweden; Division of Geriatric Medicine, Department of Clinical Sciences in Malmö, Lund University, Malmö, Skåne County, Sweden; Division of Geriatric Medicine, Department of Clinical Sciences in Malmö, Lund University, Malmö, Skåne County, Sweden; Division of Geriatric Medicine, Department of Clinical Sciences in Malmö, Lund University, Malmö, Skåne County, Sweden; Division of Geriatric Medicine, Department of Clinical Sciences in Malmö, Lund University, Malmö, Skåne County, Sweden

**Keywords:** parkinsonism, cognitive dysfunction, dementia/epidemiology, longitudinal studies, older adults

## Abstract

**Introduction:**

Cognitive impairment is common in parkinsonian disorders, but its extent among older adults with subtle motor abnormalities without a diagnosed movement disorder remains unclear. We examined cognitive decline, domain-specific cognitive function and incident dementia in older adults with subthreshold parkinsonism and parkinsonism (primary analysis). Sensitivity analyses were repeated using an additional definition of Mild Parkinsonian Signs for comparison.

**Methods:**

In the longitudinal general-population study Good Ageing in Skåne, 398 older adults aged 80–101 years (mean 86) were examined using the Unified Parkinson’s Disease Rating Scale part III and a comprehensive cognitive test battery. Participants were followed for a mean of 2.67 (0–9) years. Linear mixed models adjusted for age, sex and education were used to examine cognitive trajectories.

**Results:**

At baseline, 54.4% of participants met the criteria for subthreshold parkinsonism and 23.6% for parkinsonism. Subthreshold parkinsonism was associated with reduced performance in perceptual speed (*z*-score estimate: −0.51), language (−0.36), executive function (−0.36) and global cognition (−0.33). Parkinsonism was associated with reduced performance in memory (−0.30), perceptual speed (−0.40), language (−0.33), executive function (−0.32) and global cognition (−0.34). Memory decline was most pronounced in both groups. During follow-up, 18% of participants developed dementia; of these, 46% had subthreshold parkinsonism and 26% parkinsonism at baseline.

**Conclusion:**

Subthreshold parkinsonism and parkinsonism are associated with cognitive impairment, particularly perceptual speed, language, executive function and global cognition that progress over time. These findings support parkinsonian signs as a prodromal state and highlight the importance of early detection for dementia prevention.

## Key points

- Parkinsonian signs are associated with cognitive impairment that progresses over time.
- Parkinsonian signs might be a prodromal state and highlight the importance of early detection for dementia prevention.
- Inclusion of motor symptom assessment in cognitive evaluations may improve early identification of at-risk individuals.

## Introduction

Parkinsonian signs refer to rigidity, bradykinesia, tremor and alterations in posture and gait observed in individuals without a prior diagnosis of a movement disorder [1, 2]. In the literature, several definitions of parkinsonian signs as Mild Parkinsonian Signs (MPS) and subthreshold parkinsonism have been described, reflecting the same underlying phenomenon [3–8].

Cognitive impairment is frequently identified in individuals exhibiting parkinsonism or related motor symptoms, with clinical manifestations ranging from mild cognitive impairment (MCI) to dementia, and is associated with declines in global cognition, executive function and biomarkers indicative of neurodegeneration [9–12]. Given the established links between parkinsonian symptoms and cognitive decline, assessing cognition in individuals with parkinsonian signs is of increasing clinical importance.

Despite these implications, cognitive trajectories in older adults with parkinsonian signs remain underexplored, particularly in very old adults. To address this gap, we followed community-dwelling individuals aged 80 years and above over a 9-year period. The overarching aim was to examine cognitive impairment and decline, including domain-specific cognitive profiles, in individuals with parkinsonian signs or parkinsonism. We used definitions of subthreshold parkinsonism and parkinsonism as primary definitions and explored whether findings were consistent using a definition of MPS. The specific objectives were: (i) to determine whether individuals with subthreshold parkinsonism/MPS or parkinsonism exhibit cognitive impairment; (ii) to assess whether these individuals demonstrate deficits in specific cognitive domains (memory, perceptual speed, verbal fluency and executive functioning) compared to symptom-free counterparts; (iii) to evaluate the trajectory of cognitive decline over time; and (iv) to examine whether individuals with subthreshold parkinsonism/MPS or parkinsonism are at increased risk of developing dementia.

## Methods

### Study design

**Figure 1:**
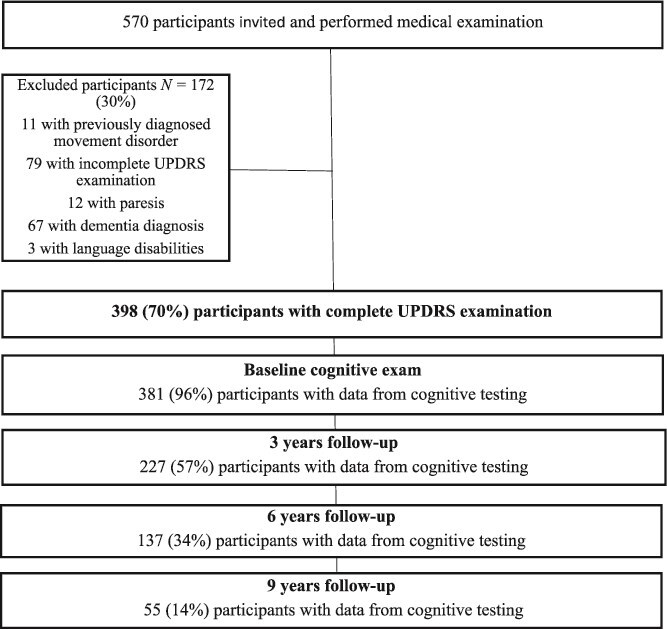
Flowchart showing the selection of study participants from the original Good Ageing in Skåne cohort. Exclusion criteria included prior diagnosis of movement disorders, incomplete UPDRS-III assessments, paresis of any origin, severe language disabilities and dementia diagnosis.

The current study was conducted as a longitudinal, population-based study utilising data from the ‘Good Ageing in Skåne’ cohort [13]. Data from 570 community-dwelling participants aged 80 years and older were collected and followed for up to 3, 6 and 9 years. According to the criteria below, 172 participants were excluded (Figure 1). Participants were randomly selected from the Swedish National Population Registry and invited by post. Both urban and rural residents of Skåne County were included. Home visits were offered to individuals unable to attend the research clinic in Malmö. Examinations were conducted by a physician, a nurse and a cognitive test administrator. Participants also completed an extensive questionnaire. Study procedures and timelines have been detailed previously [13].

### Exclusion criteria

Participants were excluded if they had a diagnosed movement disorder at baseline, an incomplete Unified Parkinson’s Disease Rating Scale (UPDRS) motor examination, paresis of any origin, diagnosis of dementia prior to examination or fulfilled criteria for dementia according to Diagnostic and Statistical Manual of Mental Disorders, Fourth Edition (DSM IV) criteria during the examination. Sensitivity analysis was performed by including participants with dementia, see Supplementary Appendix S1, Tables  S1–S3. Movement disorders were defined as Parkinson’s disease (PD), atypical parkinsonism (e.g. multiple system atrophy, progressive supranuclear palsy, corticobasal degeneration), Lewy body dementia, other degenerative basal ganglia diseases or Huntington’s disease. Information regarding paresis was obtained from the medical examination and the nurse protocol. Diagnostic data were obtained from the Swedish National Patient Registry.

### Ethical considerations

All participants provided written informed consent prior to enrolment. The study was conducted in accordance with the Declaration of Helsinki and approved by the Regional Ethical Review Board at Lund University, Sweden (LU 744-00).

### Data collection

Descriptive variables included age, sex, educational level and UPDRS part III motor scores. Education was self-reported and defined as total years of schooling. The UPDRS motor assessment was conducted by a trained physician to identify parkinsonian signs. To classify participants as having no parkinsonian signs, subthreshold parkinsonism or parkinsonism, we applied criteria of subthreshold parkinsonism by Postuma *et al*. in combination with Movement Disorder Society (MDS). Subthreshold parkinsonism was defined as having UPDRS-score ≥3 points excluding postural- and action tremor [8]. Parkinsonism was defined as bradykinesia in combination with either rest tremor, rigidity or both [14].

To allow comparison with prior literature, we also applied criteria for MPS and parkinsonism by Louis *et al*. [3]. MPS was defined as meeting one or more of the following criteria: (a) two or more UPDRS-III items scored as 1, (b) one item scored ≥2, or (c) a rest tremor item score of 1. UPDRS items were grouped into four categories: tremor, rigidity, bradykinesia and gait/balance/axial function. Parkinsonism was defined as having scores of ≥2 in at least two different categories on two or more items. Individuals classified as having parkinsonism were not included in the MPS group.

### Cognitive assessment

Cognitive testing was performed by an experienced test administrator and has been described in detail elsewhere [15]. In brief, four cognitive domains were assessed: episodic memory, perceptual speed, language and executive functioning.

Episodic memory: 16-item word recall and 16-item word recognition tests.

Perceptual speed: digit cancellation and pattern comparison tests.

Language: verbal fluency (naming animals and professions).

Executive functioning: a shortened Trail Making Test B and digit span backwards.

Raw test scores were standardised using baseline means and standard deviations. For each domain, a composite *z*-score was calculated as the mean of the two relevant test scores. If only one test was available for a domain, that domain was excluded from *z*-score calculation. Participants with at least two domain *z*-scores were assigned a global cognitive score as the average of available *z*-scores.

### Dementia diagnosis

Information on dementia diagnosis was obtained from the Swedish National Patient Registry and clinical evaluations. Dementia was categorised based on the DSM-IV. Participants who fulfilled criteria for dementia according to DSM-IV on the day of assessment were classified as having dementia. Those diagnosed prior were considered previously demented, while those diagnosed post-assessment were categorised as having developed dementia after baseline, regardless of the exact timing and type of dementia.

### Statistical analysis

All statistical analyses were performed using IBM SPSS Statistics for Windows, Version 28.0 (Armonk, NY: IBM Corp). Confidence intervals or standard errors were used to estimate precision, and statistical significance was set at *P* < .05. Raw and standardised (*z*-score) cognitive test scores were reported. To examine differences in cognitive performance between symptomatic and asymptomatic groups, analysis of covariance (ANCOVA) models were used, adjusting for age, sex and education. To further assess trends in cognitive performance, an additional general linear model was used and presented as *P*-value for trend. To investigate cognitive profiles and longitudinal decline across groups (no parkinsonian symptoms, subthreshold parkinsonism or parkinsonism), linear mixed-effects models were applied to account for repeated measures. Outcome variables were the standardised test scores. Models assessed global cognition and the four cognitive domains (perceptual speed, episodic memory, verbal fluency, executive functioning). Each model included fixed effects for age, sex, education, number of study examinations and accumulated time in the study. Longitudinal effects were assessed using an interaction term between group classification and time. A random intercept at participant level was included in all models.

The model equation was:


\begin{align*}Y_{i}=\,&\beta_{0}+\beta_{1}GroupM/P_{i}+\beta_{2}Age_{i}+\beta_{3}Sex_{i}\\&+\beta_{4}\,Time_{it}+\beta_{5}\, Examination_{it}\\&+\beta_{6}(GroupM/P_{it}\, x\,CumulativeTime_{it})+u_{i0}+ \epsilon_{it}\end{align*}

where *Y*_𝑖𝑡_ is the cognitive test score for individual _𝑖_ at time _𝑡_; *GroupM/P_𝑖_* is the categorical group variable (subthreshold parkinsonism, parkinsonism or no parkinsonian signs); *Examination_𝑖t_* indicates the test session (first, second); *u*_𝑖0_ is the random intercept (ID) and *ϵ_𝑖𝑡_* is the residual error.

To assess the risk of incident dementia during follow-up, an unadjusted Cox regression model was performed. Thereafter, an age-adjusted Fine Gray model was used to assess competing risks.

## Results

### Participant characteristics

Figure 1 displays the study flow chart. Of the 398 participants, 233 (60.1%) were female, with an age range of 80–101 years (mean 85.7). In total, 217 participants (54.5%) met the criteria for subthreshold parkinsonism and 94 (23.6%) for parkinsonism according to MDS criteria. Educational level ranged from 5 to 24 years, with a mean of 9.6 years. Regarding UPDRS-examination, total mean was 12, ranging from 0 to 42. A total of 311 individuals fulfilled the criteria for bradykinesia, 73 for rest tremor and 42 for rigidity. Further demographic and clinical characteristics are provided in Table 1. Sensitivity analysis including individuals with dementia is provided in Supplementary Appendix S1, Table  S1.

**Table 1:** Demographic and clinical characteristics of study participants, stratified by parkinsonian symptoms.

	No parkinsonian signs	Subthreshold parkinsonism	Parkinsonism	Total	*P*-value
Participants (*n*, %)	87 (21.9)	217 (54.5)	94 (23.6)	398 (100)	–
*Demographic data*					
Age, years mean (min-max)	84 (80.0–98.8)	86 (80.3–101)	86.7 (80.5–97.5)	85.7 (80.0–101)	<.001
Females (%)	45 (51.7)	138 (63.6)	52 (55.3)	233 (60.1)	.116
Males (%)	42 (48.3)	79 (36.4)	42 (44.7)	155 (39.9)	.116
Educational level, years mean (min-max)	9.8 (5.0–20.0)	9.6 (6.0–24.0)	9.58 (6.0–20.0)	9.6 (5.0–24.0)	.927
*Clinical characteristics*					
MMSE mean (min-max)	27.2 (23–30)	26.5 (20–30)	26.1 (20–30)	26.5 (20–30)	.004
UPDRS, total score mean (min-max)	1.7 (0.0–5.0)	13 (3.0–41)	18 (7–42)	12 (0.0–42)	<.001
Bradykinesia (*n*, %)	26 (30)	194 (89.4)	94 (100)	311 (80.2)	<.001
Rest tremor (*n*, %)	2 (2.3)	5 (2.3)	68 (72.3)	73 (18.8)	<.001
Rigidity (*n*, %)	1 (1.1)	5 (2.3)	36 (38.3)	42 (10.8)	<.001

Values are presented as means (min-max) or number (%). Statistical significance assessed with ANOVA to chart differences between the groups. Significance level set at 0.05. –, not available.

### Baseline global cognition and cognitive domains

Baseline global cognitive function varied significantly across groups, with individuals with parkinsonism demonstrating the lowest performance, followed by those with subthreshold parkinsonism. Detailed results are presented in Table 2. Across the cognitive test battery, participants with parkinsonism consistently scored lower than those with subthreshold parkinsonism. Perceptual speed was significantly lower in both symptomatic groups, demonstrated by reduced scores on both digit cancellation and pattern comparison tests. Verbal fluency was also lower among participants with subthreshold parkinsonism and parkinsonism. Executive function showed progressive decline across groups, with a milder reduction in digit span compared to processing speed. Memory performance differences were only statistically significant regarding word recognition performance, although free recall scores showed the greatest group variation.

**Table 2:** Baseline raw scores and standardised *z*-scores from cognitive tests by each group.

Domains and tests	No parkinsonian signs	Subthreshold parkinsonism	Parkinsonism	*F*	*P-*value	*P*-value for trend
**Memory**						
Word recall	6.5 (2.0)	6.0 (2.3)	5.5 (2.1)	2.2	.105	–
Word recognition	13.2 (2.6)	12.2 (3.0)	11.9 (3.4)	4.0	.019	–
Sum of above *z*-score	0.1 (0.9)	−0.1 (0.9)	−0.3 (1.0)	2.4	.097	.031
**Perceptual speed**						
Digit cancellation	16.8 (3.5)	14.9 (3.6)	14.9 (3.8)	7.5	<.001	–
Pattern comparison	13.1 (2.9)	11.0 (3.0)	11.0 (2.8)	13.2	<.001	–
Sum of above *z*-score	0.5 (0.8)	−0.1 (0.8)	−0.02 (0.8)	14.0	<.001	<.001
**Executive functioning**						
Trail making test B (seconds)	31.3 (14.0)	40.6 (28.2)	38.5 (14.1)	2.6	.076	–
Digit span backwards	4.1 (0.9)	3.8 (1.0)	3.7 (1.0)	1.8	.164	–
Sum of above *z*-score	0.2 (0.6)	−0.2 (0.8)	−0.1 (0.7)	4.6	.011	.062
**Language**						
Verbal fluency animals	19.3 (6.0)	16.9 (5.9)	17.1 (4.5)	4.3	.015	–
Verbal fluency occupation	13.5 (4.5)	12.5 (4.5)	11.9 (4.5)	1.8	.161	–
Sum of above *z*-score	0.2 (0.9)	−0.2 (0.9)	−0.2 (0.8)	3.4	.035	.058
**Global cognition** (aggregated measure of *z-*scores above)	0.3 (0.5)	−0.1 (−1.8 to 1.4)	−0.2 (−1.7 to 1.2)	14	<.001	<.001

Domains include memory, perceptual speed, executive function and language. Values presented as mean (SD). Statistical significance was assessed with ANCOVA analyses adjusted for age, sex and education. Trend was assessed with a general linear model. –, not available.

### Longitudinal follow-up (3, 6 and 9 years)

Linear mixed-effects model analyses revealed significant group differences across all cognitive domains between individuals with parkinsonism and those without parkinsonian symptoms. In the subthreshold parkinsonism group, significant differences were observed in perceptual speed, executive functioning, language and global cognition compared to symptom-free participants (Table 3). Global cognitive function was moderately reduced in the subthreshold parkinsonism group, while the parkinsonism group showed overall lower performance. Time-interaction effects revealed that cognitive decline in parkinsonism was significant regarding memory, executive functioning and global cognition. Domain-specific trajectories showed moderate decline in perceptual speed in subthreshold parkinsonism and a steeper decline in parkinsonism, although time effects were not statistically significant. Executive function declined significantly in parkinsonism and showed a non-significant trend in subthreshold parkinsonism. Language performance worsened in both groups, though time interaction effects were non-significant. Memory decline was significant in both parkinsonism and subthreshold parkinsonism.

**Table 3:** Linear mixed-effects model results showing estimated mean beta coefficients **β (SE)** for cognitive differences in baseline and follow-up, as well as rates of decline over time.

	Overall cognition		Cognitive decline	
Domains	Subthreshold parkinsonism	Parkinsonism	Subthreshold parkinsonism	Parkinsonism
Memory	−0.15 (0.11)	−0.30 (0.13)^*^	−0.02 (0.02)^*^	−0.01 (0.02)^*^
Perceptual speed	−0.51 (0.10)^**^	−0.40 (0.12)^**^	0.01 (0.02)	0.00 (0.03)
Executive functioning	−0.36 (0.09)^**^	−0.32 (0.10)^*^	−0.03 (0.02)	−0.08 (0.03)^*^
Language	−0.36 (0.10)^**^	−0.33 (0.12)^*^	−0.03 (0.02)	−0.05 (0.03)
Global cognition	−0.35 (0.07)^**^	−0.34 (0.09)^**^	−0.01 (0.01)	−0.04 (0.02)^*^

The model was adjusted for age, sex and education. Reference group: no parkinsonian signs. Significance level set at 0.05 (^*^P ≤ .05; ^**^P ≤ .001).

**Figure 2:**
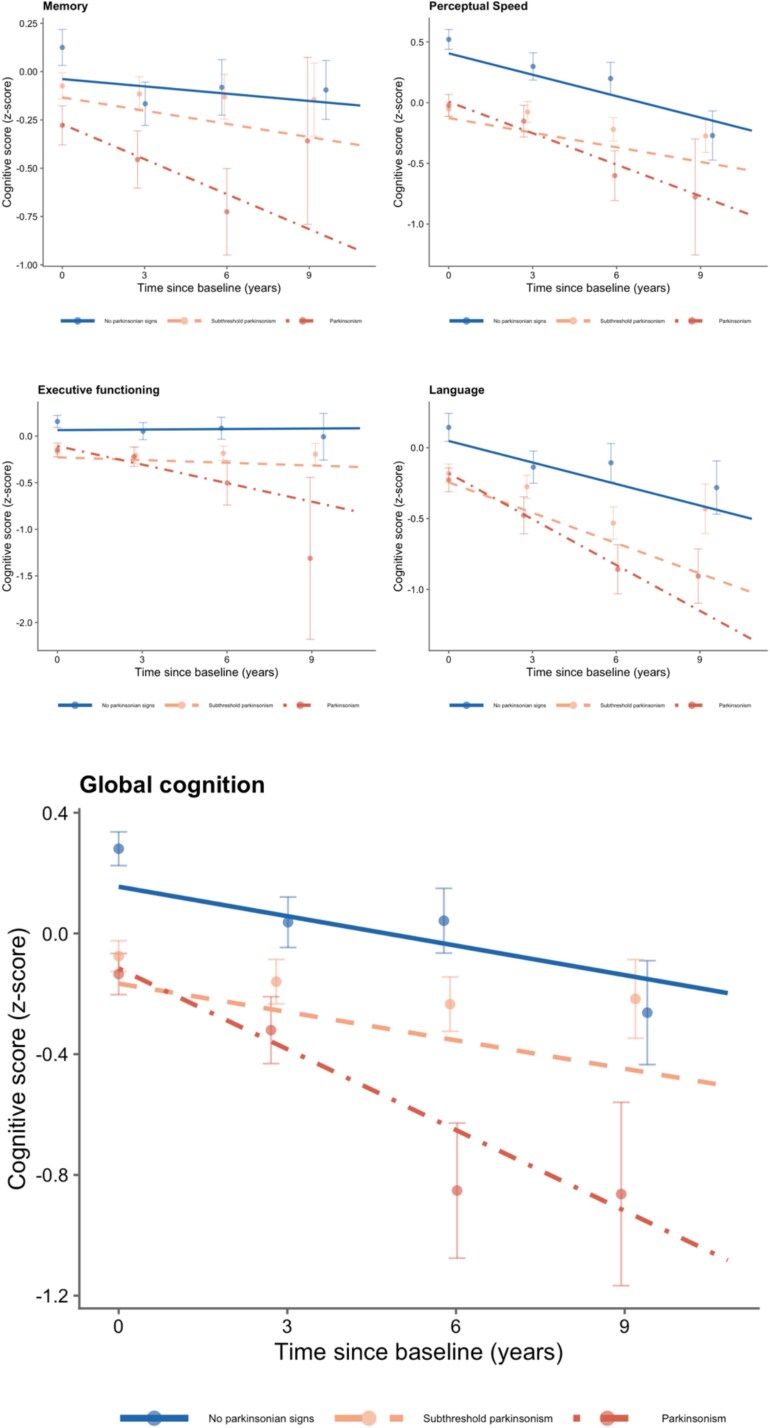
Predicted cognitive trajectories (lines) and observed group means ± SE during follow-up (points ± error bars) for five cognitive domains. No parkinsonian signs (lines), subthreshold parkinsonism (error bars), parkinsonism (error bars + points). Continuous covariates held at sample mean for fixed-effect predictions.

Pairwise comparisons (see Supplementary Appendix S1, Table  S3B) confirmed significant group differences. Individuals with parkinsonism had lower outcomes in perceptual speed, executive function, language and global cognition compared to symptom-free individuals. The subthreshold parkinsonism group scored significantly lower in perceptual speed, executive function and language compared to the symptom-free group. Comparisons between subthreshold parkinsonism and parkinsonism groups showed some differences across all cognitive domains, even though they were not statistically significant. For an illustration of cognitive trajectories, see Figure 2.

### Sensitivity analysis of mild parkinsonian signs

Baseline global cognitive function varied significantly across groups, with individuals with parkinsonism demonstrating the lowest performance and the highest scores observed among participants without parkinsonian signs. Across cognitive domains, perceptual speed showed marked reduced results (see Supplementary Appendix S1, Table  S2X).

Linear mixed-effects model analyses revealed significant group differences across all cognitive domains between individuals with parkinsonism and those without parkinsonian signs. Participants with MPS also showed significantly lower performance in perceptual speed, executive function, language and global cognition compared with symptom-free participants (see Supplementary Appendix S1, Table  S3AX). In contrast to subthreshold parkinsonism, pairwise analyses between MPS and parkinsonism showed significant differences across all cognitive domains.

### Dementia outcomes during follow-up

During the follow-up period, 74 participants (18%) received a new diagnosis of dementia. Of these, 26% (*n* = 19) had parkinsonism at baseline and 46% (*n* = 34) had subthreshold parkinsonism. The unadjusted standard Cox model (time since baseline) showed no statistically significant group differences, though hazard ratio in the parkinsonism group was 1.6 (95% CI 0.88–2.92, *P* = .126) (see Supplementary Appendix S1, Table  4.1). After adjusting for age, the subdistribution hazard ratio (SHR) for subthreshold parkinsonism was 0.62 (*P* = .075) and the parkinsonism group SHR was ~1.0 (*P* = .99). Results from sensitivity analyses that included individuals with dementia at baseline are provided in Supplementary Appendix S1.

## Discussion

In this longitudinal population-based study of adults over 80 years of age, individuals with subthreshold parkinsonism or parkinsonism exhibited significantly impaired cognitive performance at baseline and over time compared to symptom-free individuals. Deficits were most pronounced in perceptual speed, executive function, language and global cognition. Findings were consistent in sensitivity analysis of MPS and when including individuals with dementia. These findings suggest that, even in the absence of a formal PD diagnosis, cognitive impairment in individuals with parkinsonian signs mirrors aspects of cognitive decline seen in other parkinsonian syndromes.

We did not observe differences in incidence of dementia between groups during follow-up. However, the risk was directionally higher in the parkinsonism group. These findings do not support an association between parkinsonian signs and risk of dementia. This might reflect underreporting of dementia diagnoses and mortality-related attrition in this cohort of the oldest-old.

We analysed parkinsonian signs using two different definitions. The primary analyses used the subthreshold parkinsonism by Postuma *et al*. in combination with the definition of parkinsonism by MDS for improved generalisability. Sensitivity analysis with the frequently used definition of MPS and parkinsonism by Louis *et al*. presented similar results. This indicates that both definitions are similar and may be used interchangeably. Furthermore, cognitive differences between subthreshold parkinsonism and MDS-defined parkinsonism were relatively small, likely reflecting phenotypic reclassification rather than a pure increase in overall severity. This may relate to the high prevalence of bradykinesia and heterogeneous expression of rigidity, rest tremor and axial features in population-based samples at an advanced age. In contrast, Louis-defined MPS may identify individuals with milder motor involvement and clearer separation from parkinsonism, potentially amplifying cognitive differences between groups.

In the main analysis, we excluded individuals with dementia. Exclusion of these participants could have restricted the study’s external validity and masked the natural variation seen in real-world ageing populations [16]. We included baseline dementia cases in sensitivity analyses to evaluate whether the observed longitudinal patterns were robust across the full spectrum of cognitive impairment, and to assess potential selection bias introduced by restricting the cohort. Similar results support generalisability of the findings to very old populations, whereas attenuation would be consistent with floor effects and differential attrition among participants with dementia.

Cognitive dysfunction is a core feature across parkinsonian syndromes. In PD, cognitive decline is typically gradual, with executive and visuospatial deficits appearing early [12, 17]. Our findings are consistent with this heterogeneity. Previous studies demonstrated that motor symptoms in autopsy-confirmed Alzheimer’s disease (AD) predicted progression to severe cognitive impairment. Similarly, progression of parkinsonian signs increased the risk of incident AD [18, 19]. A meta-analysis highlighted that movement disorders predicted reduced survival in AD patients [20]. Although our study focused on cognitive trajectories, the cognitive domains affected often overlap with those linked to mortality risk. Furthermore, bradykinesia was linked to poorer balance and functional outcomes, consistent with perceptual and motor slowing as early markers, while rigidity also predicted adverse outcomes [21, 22].

This is in line with our mixed model findings. As motor symptoms do not occur in isolation, previous studies [23–25] found that patients with dementia had a higher burden of geriatric syndromes interacting with motor and cognitive decline, potentially accelerating the trajectory we observed in our cohort. Subtle parkinsonian signs were linked with reduced synaptic density across multiple brain regions [26], independent of atrophy or white matter lesions. Importantly, early motor signs in identifying high-risk individuals are further supported by the diagnostic value of a simplified five-item UPDRS-based scale in detecting parkinsonism among individuals with MCI [27], particularly those at risk for dementia with Lewy bodies (DLB). These findings reinforce that parkinsonian signs are not benign features of ageing.

Strengths of this study include its population-based design, the inclusion of very old adults, a group often underrepresented in neurodegenerative research and the use of linear mixed-effects models which account for repeated measures and missing data. However, limitations should be acknowledged. Attrition over time may have introduced selection bias and reduced statistical power in later follow-ups. We did not adjust for potential confounders such as depressive symptoms, vascular comorbidities or medication use. Although UPDRS-based classification was used, full clinical diagnostic criteria for PD could not be applied, introducing some diagnostic uncertainty. The comparison group was small and likely represented a particularly robust subset, which may have inflated group differences. Additionally, many participants had cognitive decline at the time of subthreshold parkinsonism or parkinsonism classification; therefore, caution is warranted in inferring directionality.

## Conclusion

This study demonstrates that both subthreshold parkinsonism (or MPS) and parkinsonism are associated with poorer cognitive performance in very old adults, particularly affecting perceptual speed, language, executive function and global cognition. These signs also predict longitudinal cognitive decline, independent of baseline dementia.

Our findings suggest that parkinsonian signs represent a clinically relevant prodromal state in the spectrum of cognitive ageing and neurodegeneration. The inclusion of motor symptom assessment in cognitive evaluations may improve early identification of individuals at risk and intervention in preventing or delaying progression of dementia.

## Supplementary Material

aa-25-3424-File002_afag160

## Data Availability

Data can be available upon request.
