# Supplementary material for: Parkinsonian signs and cognitive trajectories in older adults: a population-based longitudinal study
Source: Age Ageing. 2026 Jun 8;55(6):afag160. doi: 10.1093/ageing/afag160 (PMC13245726; doi:10.1093/ageing/afag160)
Supplement: aa-25-3424-File002_afag160 [file aa-25-3424-file002_afag160.docx]

**APPENDIX**

Parkinsonian Signs and Cognitive Trajectories in Older Adults:

A population-Based Longitudinal Study

Supplementary results that contain individuals with dementia together with sensitivity analyses of MPS and parkinsonism according to criteria by Louis et al.

**Table of contents**

1. *Supplementary table of Pairwise comparisons*
2. *Sensitivity analysis containing individuals with dementia*
   1. Supplementary table S1
   2. Supplementary table S2
   3. Supplementary table S3A
   4. Supplementary table S3B
3. *Sensitivity analysis* *of MPS and parkinsonism according to criteria by Louis et al.*
   1. Supplementary table S1X
   2. Supplementary table S2X
   3. Supplementary table S3AX
   4. Supplementary table S3BX
4. *Incident dementia – standard Cox regression*
5. *Competing-risks analysis*
6. *Attrition and mortality*
   1. Attrition at year 3 and year 6
7. **Supplementary table of Pairwise comparisons**

**Supplementary table 3B.** Pairwise comparisons between groups from the mixed model analysis adjusted for age, gender and educational level. Data presented as mean differences in standardised aggregated z-scores (SE), as in table 2. Significance level 0.05 (* = p ≤0.05; ** = p ≤ 0.001).

|  | Subthreshold parkinsonism compared with | Parkinsonism compared with | |
| --- | --- | --- | --- |
| Domains | No parkinsonian signs | No parkinsonian signs | Subthreshold parkinsonism |
|  |  |  |  |
| Memory | -0.19 (0.11) | -0.40 (0.13)* | -0.21 (0.12) |
| Perceptual Speed | -0.58 (0.10)** | -0.54 (0.13)** | 0.04 (0.11) |
| Executive functioning | -0.39 (0.09)** | -0.43 (0.11)** | -0.04 (0.10) |
| Language | -0.42 (0.11)** | -0.45 (0.13)** | -0.02 (0.11) |
| Global cognition | -0.41 (0.07)** | -0.46 (0.09)** | -0.05 (0.08) |

|  | No parkinsonian signs | Subthreshold parkinsonism | Parkinsonism | Total | P-value |
| --- | --- | --- | --- | --- | --- |
| Participants (n, %) | 92 (19.9) | 259 (55.9) | 112 (24.2) | 463 (100) |  |
| *Demographic data* |  |  |  |  |  |
| Age (yrs.) | 84.1 (80 – 98) | 86.3 (80–101) | 86.7 (80–97) | 85.9 (80-101) | <0.001 |
| Females (%) | 47 (51.1) | 164 (63.3) | 60 (53.6) | 271 (58.5) | 0.58 |
| Males (%) | 45 (48.9) | 95 (36.7) | 52 (53.6) | 192 (41.5) | 0.58 |
| Educational level (yrs.) | 9.8 (5-20) | 9.4 (6-24) | 9.3 (4-20) | 9.5 (4-24) | 0.643 |
| *Clinical characteristics* |  |  |  |  |  |
| MMSE  mean (min-max) | 26.8 (14-30) | 25.4 (11-30) | 25.2 (8-30) | 25.6 (8-30) | 0.002 |
| UPDRS, total score  mean (min-max) | 1.8 (0-6) | 13.8 (3-45) | 18.7 (7-42) | 12.6 (0-45) | <0.001 |
| Bradykinesia (n, %) | 27 (29.3) | 225 (86.9) | 112 (100) | 364 (78.6) | <0.001 |
| Rest tremor (n, %) | 1 (1.1) | 7 (2.7) | 80 (71.4) | 88 (19.0) | <0.001 |
| Rigidity (n, %) | 1 (1.1) | 5 (1.9) | 44 (39.3) | 50 (10.8) | <0.001 |
| Diagnosis of dementia prior to study examination (n, %) | 1 (1.1) | 16 (6.2) | 7 (6.2) | 24 (5.2) |  |
| Dementia according to DSM IV on the day of study examination  (n, %) | 4 (4.3) | 26 (10.0) | 11 (9.8) | 41 (8.8) |  |
| Total Dementia (n, %) | 5 (5.4) | 42 (16.2) | 18 (16.0) | 65 (14.0) | 0.04 |

1. **Sensitivity analysis containing individuals with dementia**

**2.1 Supplementary table S1.**  Demographic and clinical characteristics of study participants, stratified by parkinsonian signs. Values are presented as means (min-max) or number (%). Statistical significance assessed with ANOVA to chart differences between the groups. Significance level 0.05.

**2.2 Supplementary table S2.** Baseline raw scores and standardised z-scores from cognitive tests by each group. Domains include memory, perceptual speed, executive function, and language. Values presented as mean (SD). Statistical significance was assessed with ANCOVA analyses adjusted for age, sex, and education.

| Domains and tests | No parkinsonian signs | Subthreshold parkinsonism | Parkinsonism | F | P-value |
| --- | --- | --- | --- | --- | --- |
| **Memory** |  |  |  |  |  |
| Word recall | 6.3 (2.2) | 5.6 (2.4) | 5.3 (2.2) | 2.37 | 0.094 |
| Word recognition | 13.1 (2.7) | 11.9 (3.4) | 11.8 (3.5) | 4.02 | 0.019 |
| Sum of above  *Z-score* | 0.04 (1.0) | -0.24 (1.0) | -0.4 (1.0) | 2.17 | 0.116 |
| **Perceptual Speed** |  |  |  |  |  |
| Digit cancellation | 16.8 (3.5) | 14.6 (3.6) | 14.6 (4.1) | 9.52 | <0.001 |
| Pattern comparison | 13.0 (3.0) | 10.6 (3.1) | 10.6 (3.1) | 15.5 | <0.001 |
| Sum of above  *Z-score* | 0.5 (0.8) | -0.1 (0.8) | -0.2 (0.9) | 16.9 | <0.001 |
| **Executive functioning** |  |  |  |  |  |
| Trail making test B (seconds) | 31.3 (13.8) | 41.6 (28.8) | 40.1 (19.4) | 3.21 | 0.042 |
| Digit span backwards | 4.0 (0.9) | 3.7 (1.0) | 3.7 (1.0) | 3.34 | 0.037 |
| Sum of above  *Z-score* | 0.2 (0.6) | -0.3 (0.8) | -0.2 (0.7) | 7.37 | <0.001 |
| **Language** |  |  |  |  |  |
| Verbal fluency  animals | 18.9 (6.2) | 16.3 (6.2) | 16.3 (5.1) | 5.34 | 0.005 |
| Verbal fluency occupation | 13.2 (21.7) | 12.0 (4.7) | 11.3 (4.7) | 2.24 | 0.108 |
| Sum of above  *Z-score* | 0.1 (1.0) | -0.3 (1.0) | -0.4 (0.9) | 4.46 | 0.012 |
| **Global cognition** (aggregated measure of *Z-scores above*) | 0.2 (0.6) | -0.2 (0.7) | -0.3 (0.6) | 12.6 | <0.001 |

**2.3 Supplementary table S3A.** Linear mixed-effects model results showing estimated mean beta coefficients **(β; SE)** for cognitive differences and rates of decline over time. Adjusted for age, sex, and education. Reference group: no parkinsonian signs. Significance level 0.05 (* = p ≤0.05; ** = p ≤ 0.001).

|  | Overall cognition | | Cognitive decline | |
| --- | --- | --- | --- | --- |
| Domains | Subthreshold parkinsonism | Parkinsonism | Subthreshold parkinsonism | Parkinsonism |
| Memory | -0.19 (0.11) | -0.28 (0.13)* | -0.001 (0.02) | -0.043 (0.03) |
| Perceptual Speed | -0.56 (0.10)** | -0.53 (0.12)** | 0.02 (0.02) | -0.02 (0.03) |
| Executive functioning | -0.38 (0.09)** | -0.31 (0.10)* | -0.41 (0.09)** | -0.30 (0.11)* |
| Language | -0.38 (0.11)** | -0.38 (0.13)** | -0.02 (0.02) | -0.05 (0.03) |
| Global cognition | -0.39 (0.08)** | -0.36 (0.09)** | -0.43 (0.08)** | -0.38 (0.09)* |

**2.4 Supplementary table S3B.** Pairwise comparisons between groups from the mixed model analysis adjusted for age, gender and educational level. Data presented as mean differences in standardised aggregated z-scores (SE), as in table 2. Significance level 0.05 (* = p ≤0.05; ** = p ≤ 0.001).

|  | Subthreshold parkinsonism compared with | Parkinsonism compared with | |
| --- | --- | --- | --- |
| Domains | No parkinsonian signs | No parkinsonian signs | Subthreshold parkinsonism |
| Memory | -0.23 (0.12) | -0.40 (0.14)* | -0.18 (0.12) |
| Perceptual Speed | -0.63 (0.11)** | -0.69 (0.13)** | -0.06 (0.11) |
| Executive functioning | -0.41 (0.09)** | -0.39 (0.11)** | 0.02 (0.09) |
| Language | -0.44 (0.11)** | -0.50 (0.13)** | -0.06 (0.11) |
| Global cognition | -0.44 (0.08)** | -0.48 (0.09)** | -0.04 (0.08) |

1. **Sensitivity analysis of Mild Parkinsonian Signs and parkinsonism according to criteria by Louis et al.**

**3.1 Supplementary table S1X.**  Demographic and clinical characteristics of study participants, stratified by parkinsonian signs. Values are presented as means (min-max) or number (%). Statistical significance assessed with ANOVA to chart differences between the groups. Significance level 0.05.

|  | No parkinsonian signs | MPS | Parkinsonism | Total | P-value |
| --- | --- | --- | --- | --- | --- |
| Participants (n, %) | 41 (10) | 243 (61) | 114 (29) | 398 (100) |  |
| *Demographic data* |  |  |  |  |  |
| Age, years  mean (min-max) | 84.2 (80 – 92) | 85.1 (80 – 99) | 87.8 (81 – 101) | 85.8 (80 – 101) | <0.001 |
| Females (%) | 23 (56.1) | 139 (57.2) | 73 (64) | 235 (59) | 0.437 |
| Males (%) | 18 (43.9) | 104 (42.8) | 41 (36) | 163 (41) | 0.437 |
| Educational level, years  mean (min-max) | 10.3 (5-20) | 9.6 (6-20) | 9.7 (6-24) | 9.7 (5-24) | 0.456 |
| *Clinical characteristics* |  |  |  |  |  |
| MMSE  mean (min-max) | 27.7 (24-30) | 26.6 (20-30) | 26.1 (20-30) | 26.5 (20-30) | <0.001 |
| UPDRS, total score  mean (min-max) | 0.46 (0-1) | 9.12 (2-25) | 21.2 (5-42) | 11.7 (00-42) | <0.001 |
| Bradykinesia (n, %) | 3 (7.3) | 206 (84.8) | 105 (92.1) | 314 (78.9) | <0.001 |
| Rest tremor (n, %) | 0 (0) | 40 (16.5) | 33 (28.9) | 73 (18.3) | <0.001 |
| Rigidity (n, %) | 0 (0) | 21 (8.6) | 21 (18.4) | 42 (10.6) | <0.001 |
|  |  |  |  |  |  |

**3.2 Supplementary table S2X.** Baseline raw scores and standardised z-scores from cognitive tests by each group. Domains include memory, perceptual speed, executive function, and language. Values presented as mean (SD). Statistical significance was assessed with ANCOVA analyses adjusted for age, sex, and education.

| Domains and tests | No parkinsonian signs | MPS | Parkinsonism | F | P-value |
| --- | --- | --- | --- | --- | --- |
| **Memory** |  |  |  |  |  |
| Word recall | 6.95 (1.78) | 6.0 (2.06) | 5.51 (2.51) | 3.7 | 0.025 |
| Word recognition | 13.2 (2.2) | 12.6 (2.9) | 11.7 (3.4) | 3.6 | 0.028 |
| Sum of above  *Z-score* | 0.31 (0.8) | -0.05 (0.9) | -0.28 (1.1) | 3.7 | 0.026 |
| **Perceptual Speed** |  |  |  |  |  |
| Digit cancellation | 17.2 (2.9) | 15.6 (3.6) | 14.0 (3.9) | 8.8 | <0.001 |
| Pattern comparison | 13.5 (2.5) | 11.6 (2.9) | 10.4 (3.2) | 10.4 | <0.001 |
| Sum of above  *Z-score* | 0.62 (0.64) | 0.14 (0.75) | -0.25 (0.9) | 12.7 | <0.001 |
| **Executive functioning** |  |  |  |  |  |
| Trail making test B (seconds) | 31.2 (15.7) | 36.3 (24.4) | 44.5 (20.2) | 2.0 | 0.139 |
| Digit span backwards | 3.9 (0.8) | 3.9 (1.0) | 3.7 (1.0) | 0.4 | 0.683 |
| Sum of above  *Z-score* | 0.1 (0.6) | -0.04 (0.8) | -0.3 (0.8) | 1.4 | 0.242 |
| **Language** |  |  |  |  |  |
| Verbal fluency  animals | 19.9 (6.9) | 17.9 (5.2) | 15.8 (5.7) | 4.8 | 0.009 |
| Verbal fluency occupation | 14.3 (4.2) | 12.9 (4.5) | 11.2 (4.4) | 6.6 | 0.002 |
| Sum of above  *Z-score* | 0.29 (1.0) | -0.05 (0.9) | -0.44 (0.92) | 7.1 | <0.001 |
| **Global cognition** (aggregated measure of *Z-scores above*) | 0.33 (0.51) | -0.008 (0.54) | -0.30 (0.69) | 12.0 | <0.001 |

**3.3 Supplementary table S3AX.** Linear mixed-effects model results showing estimated mean beta coefficients **(β; SE)** for cognitive differences and rates of decline over time. Adjusted for age, sex, and education. Reference group: no parkinsonian signs. Significance level 0.05 (* = p ≤0.05; ** = p ≤ 0.001).

|  | Overall cognition | | Cognitive decline | |
| --- | --- | --- | --- | --- |
| Domains | MPS | Parkinsonism | MPS | Parkinsonism |
| Memory | -0.23 (0.14) | -0.42 (0.16)* | -0.00 (0.02) | -0.05 (0.03) |
| Perceptual Speed | -0.44 (0.13)** | -0.74 (0.14)** | 0.00 (0.02) | 0.00 (0.03) |
| Executive functioning | -0.32 (0.11)* | -0.50 (0.80)* | -0.06 (0.03)* | -0.01 (0.03)** |
| Language | -0.41 (0.13)* | -0.70 (0.15)** | -0.04 (0.02) | -0.06 (0.03)* |
| Global cognition | -0.35 (0.09)** | -0.57 (0.10)** | -0.02 (0.02) | -0.05 (0.02)* |

**3.4 Supplementary table S3BX.** Pairwise comparisons between groups from the mixed model analysis adjusted for age, gender and educational level. Data presented as mean differences in standardised aggregated z-scores (SE), as in table 2. Significance level 0.05 (* = p ≤0.05; ** = p ≤ 0.001).

|  | MPS compared with | Parkinsonism compared with | |
| --- | --- | --- | --- |
| Domains | No parkinsonian signs | No parkinsonian signs | MPS |
| Memory | -0.23 (0.14) | -0.56 (0.16)** | -0.34 (0.11)* |
| Perceptual Speed | -0.45 (0.13)** | -0.88 (0.15)** | -0.42 (0.10)** |
| Executive functioning | -0.31 (0.11)* | -0.61 (0.13)** | -0.30 (0.09)* |
| Language | -0.42 (0.13)* | -0.82 (0.15)** | -0.40 (0.10)** |
| Global cognition | -0.36 (0.10)** | -0.71 (0.11)** | -0.35 (0.07)** |

1. **Incident dementia – standard Cox regression**

A total of 78 incident dementia cases were observed in the analytic cohort. The unadjusted standard Cox model (time since baseline) showed no statistically significant group differences, though the direction was consistent with higher risk in the Parkinsonism group (HR 1.6, 95% CI 0.88–2.92, p = 0.126).

**Table 4.1**: Standard Cox model — Hazard Ratios for incident dementia (unadjusted; reference: Healthy group).

| Comparison | *HR* | *95% CI low* | *95% CI high* | *P-value* |
| --- | --- | --- | --- | --- |
| Subthreshold parkinsonism | 0.82 | 0.48 | 1.40 | 0.471 |
| Parkinsonism | 1.60 | 0.88 | 2.92 | 0.126 |

The proportional hazards assumption was not violated (global Schoenfeld test p = 0.441)

1. **Competing-risks analysis**

### Unadjusted Fine-Gray model

**Table 5.1**: Fine-Gray subdistribution hazard ratios (SHR) — unadjusted (reference: Healthy group). Death treated as competing event.

| Comparison | *SHR* | *95% CI low* | *95% CI high* | *P-value* |
| --- | --- | --- | --- | --- |
| Subthreshold parkinsonism | 0.55 | 0.33 | 0.94 | 0.027 |
| Parkinsonism | 0.85 | 0.47 | 1.54 | 0.600 |

The unadjusted Fine-Gray model shows that the Subthreshold parkinsonism group had a significantly lower subdistribution hazard than the Healthy group (SHR 0.55, p = 0.027).

### Age-adjusted Fine-Gray model

**Table 5.2**: Fine-Gray subdistribution hazard ratios (SHR) — adjusted for age at baseline examination (reference: Healthy group).

| Comparison | *SHR* | *95% CI low* | *95% CI high* | *P-value* |
| --- | --- | --- | --- | --- |
| Subthreshold parkinsonism | 0.62 | 0.36 | 1.05 | 0.075 |
| Parkinsonism | 0.99 | 0.54 | 1.83 | 0.990 |
| alder | 0.94 | 0.89 | 1.00 | 0.046 |

After adjusting for age, the Subthreshold parkinsonism SHR attenuates toward null (SHR 0.62, p = 0.075) and the Parkinsonism group SHR is approximately 1.0 (SHR 0.99, p = 0.99). Age itself is significantly associated with lower realised dementia incidence (SHR 0.94, p = 0.046 per year).

## Model Comparison Summary

**Table 5.3**: Summary of estimates across all analytical approaches. HR = Hazard Ratio (Cox); SHR = Subdistribution Hazard Ratio (Fine-Gray). Reference category: Healthy group.

| Comparison | *Standard Cox HR* | *FG SHR (unadj.)* | *FG SHR (age-adj.)* |
| --- | --- | --- | --- |
| Subthreshold parkinsonism | 0.82 (p=0.471) | 0.55 (p=0.027) | 0.62 (p=0.075) |
| Parkinsonism | 1.60 (p=0.126) | 0.85 (p=0.6) | 0.99 (p=0.99) |

All four models tell a consistent story. The nominally elevated HR for Parkinsonism in the unadjusted standard Cox model (HR 1.6) is substantially attenuated once age and competing mortality are properly addressed. The “protective” Subthreshold SHR in the unadjusted Fine-Gray is an artefact of differential mortality rather than a genuine biological effect.

1. **Attrition and mortality**

### 6.1 Attrition at year 3 - and year 6

Overall, 56.8% of participants remained active at year 3, with death accounting for 17.9% of attrition and alive-dropout for 25.3%. By year 6, death (40.4%) had overtaken alive-dropout (30.3%) as the leading reason for non-participation, with only 29.3% of the original cohort still active. Attrition patterns differed significantly by parkinsonism group at both timepoints (year-3 ² p < 0.001; year-6 ² p < 0.001).

**Table 6.1**: Year-3 attrition by parkinsonism group, n (% within group).

| Group | *Active at year 3* | *Died before year 3* | *Lost (alive) before year 3* |
| --- | --- | --- | --- |
| Healthy | 73 (84.9%) | 6 (7.0%) | 7 (8.1%) |
| Parkinsonism | 42 (44.7%) | 19 (20.2%) | 33 (35.1%) |
| Subthreshold parkinsonism | 110 (50.9%) | 46 (21.3%) | 60 (27.8%) |

Table 6.2: Year-6 attrition by parkinsonism group, n (% within group).

| Group | *Active at year 6* | *Died before year 6* | *Lost (alive) before year 6* |
| --- | --- | --- | --- |
| Healthy | 44 (51.2%) | 16 (18.6%) | 26 (30.2%) |
| Parkinsonism | 21 (22.3%) | 51 (54.3%) | 22 (23.4%) |
| Subthreshold parkinsonism | 51 (23.6%) | 93 (43.1%) | 72 (33.3%) |

**Study attrition at year 3 and year 6 by parkinsonism group**

GÅS study − n = 396 (Cox cohort)

Figure 3: Stacked bar chart of attrition status at year 3 and year 6 by parkinsonism group. Proportions within each group are shown; labels are omitted for cells < 5%.

m

**%**

**85**

**7**

**%**

**%**

**8**

**51**

**%**

**%**

**19**

**%**

**30**

**%**

**45**

**20**

**%**

**%**

**35**

**22**

**%**

**54**

**%**

**23**

**%**

**51**

**%**

**21**

**%**

**28**

**%**

**24**

**%**

**43**

**%**

**%**

**33**

**Healthy**

**Parkinsonism**

**Subthreshold parkinsonism**

0

%

25

%

50

%

75

%

100

%

Proportion of group (%)

Year 3 Year 6 Year 3 Year 6 Year 3 Year 6

Status Lost (alive) Died Active
